# Face Distortion Aftereffects in Personally Familiar, Famous, and Unfamiliar Faces

**DOI:** 10.3389/fpsyg.2012.00258

**Published:** 2012-08-01

**Authors:** Billy Ronald Peter Walton, Peter James Hills

**Affiliations:** ^1^Department of Psychology, Anglia Ruskin UniversityCambridge, UK

**Keywords:** adaptation effects, face distortion aftereffects, face perception, personally familiar versus unfamiliar faces

## Abstract

The internal face prototype is thought to be a construction of the average of every previously viewed face (Schwaninger et al., 2003). However, the influence of the most frequently encountered faces (i.e., personally familiar faces) has been generally understated. The current research explored the face distortion aftereffect in unfamiliar, famous, and personally familiar (each subject’s parent) faces. Forty-eight adult participants reported whether faces were distorted or not (distorted by shifting the eyes in the vertical axis) of a series of images that included unfamiliar, famous, and personally familiar faces. The number of faces perceived to be “odd” was measured pre- and post-adaptation to the most extreme distortion. Participants were adapted to either an unfamiliar, famous, or personally familiar face. The results indicate that adaptation transferred from unfamiliar faces to personally familiar faces more so than the converse and aftereffects did not transfer from famous faces to unfamiliar faces. These results are indicative of representation differences between unfamiliar, famous, and personally familiar faces, whereby personally familiar faces share representations of both unfamiliar and famous faces.

## Face Distortion Aftereffects in Personally Familiar and Unfamiliar Faces

Face aftereffects have been looked at extensively over the past decade since Webster and MacLin’s (1999) pioneering study. To date these aftereffects have only been tested using unfamiliar or famous faces (e.g., Zhao and Chubb, 2001; Carbon and Leder, 2005, 2006; Hills et al., 2010b). It has been found that some properties of the face aftereffects change as a function of familiarity (see Jiang et al., 2007). The present research aimed to explore the face distortion aftereffect (FDAE) using stimuli even more familiar to participants than famous people; personally familiar faces. We compared the FDAE in faces of participants’ parents with unfamiliar and famous faces to establish whether these highly familiar visual representations were affected in a similar way. A brief overview will be presented highlighting (1) the processes involved during face recognition, (2) the FDAE in unfamiliar and familiar faces, and finally (3) why personally familiar faces are especially important in the study of adaptation effects.

### Face perception and recognition

Neural substrates found in a wide variety of brain areas are involved in face processing (Taylor et al., 2009) and enable humans an unrivaled expert ability to tell apart the small differences between countless unfamiliar faces even though as a visual pattern they are very similar. For recognition to occur, however, the visual percept needs to be matched to a stored representation in memory (Bruce and Young, 1986). Faces are coded and stored in terms of both their configural and featural information (Cabeza and Kato, 2000; Mondloch et al., 2002; Leder and Carbon, 2006) along with a number of other attributes including name (Bauer, 1984), personality traits (Fiske, 1995), voice (Kriegstein et al., 2005), and emotional responses experienced while perceiving an individual (Leibenluft et al., 2004). How personally familiar one is with a particular face determines how many attributes are stored and how robust the representation is (Burton et al., 1999a). As more attributes are stored with a face, the more brain regions become involved in coding that face (Eger et al., 2005).

In order to recognize a face from different angles (Zhao et al., 2003), different distances (Wallis and Rolls, 1997), and at different times in the day (Chen et al., 2006) it follows that the stored representation must be invariant to these differences (Bruce, 1994). Faces are constantly changing due to factors such as hairstyle, age, facial expression, and weight but regardless of this they are still recognizable even years later (Bahrick et al., 1975). Familiar faces can be recognized from minimal information and even from low quality video images (Burton et al., 1999b). Unfamiliar faces, on the other hand are difficult to recognize even under optimal conditions (Kemp et al., 1997). Invariance to image changes when recognizing familiar faces suggest that their representation involves a more robust and potentially three-dimensional one than the more pictorial representation of unfamiliar faces (Ryu and Chaudhuri, 2006).

Valentine (1991) argued that face recognition is achieved by comparing all faces to a prototype that has formed as an average of all faces perceived over a lifetime (Schwaninger et al., 2003). All faces (both familiar and unfamiliar) are thought to be coded in terms of how far they deviate from this prototype or norm. Norm-based encoding has gained much empirical support over the past decade (Leopold et al., 2001). It is thought that this prototype needs to have both flexibility and stability to cope with the demands that facial recognition requires. It needs to be flexible enough to be able to recognize a face that has not been seen for a while and therefore undergone some changes such as a change in hair style, as well as stable enough to still be able to distinguish one familiar face from another.

### The face distortion aftereffect

Frisby (1979) has argued that adaptation is the psychophysicists’ microelectrode as it made it possible to probe neural response properties without the need for direct brain recording. The process has been attributed to sensory neurons becoming excited, with their responses decreasing as they become habituated. If another stimulus is subsequently perceived, perceptual distortions occur, normally causing a contrastive aftereffect. Substantial research into the aftereffects of low-level stimuli, for example orientation perception (Gibson and Radner, 1937), color and contrast perception (Blakemore and Campbell, 1969), and spatial frequency perception (Blakemore and Sutton, 1969), have been well documented. More recently, adaptation has been shown to be helpful in understanding the coding mechanisms for higher-level stimuli such as geometric shapes (Suzuki and Cavanagh, 1998), body shape (Troje et al., 2006), and faces (e.g., Leopold et al., 2001).

Webster and MacLin (1999) conducted a seminal study using faces as adapting and test stimuli. They asked participants to rate unfamiliar faces, both before and after being adapted to a distorted facial image. They found that following adaptation to a distorted face (for example, compressed), the participants perceived undistorted faces to be distorted in the opposite direction (for example, expanded). This is the crux of the FDAE. These aftereffects transferred to other unfamiliar faces and appeared to transfer to inverted faces as well. Given that the aftereffects transferred across stimuli so readily, these authors suggested the aftereffect represented the way in which faces were coded.

Since Webster and MacLin’s (1999) study, further evidence for the non-retinotopic and high-level locus for this FDAE has been found. Zhao and Chubb (2001) found that, although FDAEs were stronger when the adaptor and test images were the same size, a significant aftereffect was observed even when one image was four times larger than the other image. This highlights the size-invariance of the FDAE, suggesting that it is a higher-level phenomenon. Yamashita et al. (2005) have shown that the FDAE is resistant to a number of transformations (such as color changes and photographic negation).

Face distortion aftereffects, in unfamiliar and familiar faces, have a number of similarities and differences. Firstly even though recognition of unfamiliar faces is known to be viewpoint dependant while familiar face recognition is viewpoint-invariant (Bruce and Young, 1986) there is a debate on whether FDAEs transfer across viewpoints. Benton et al. (2006) found using unfamiliar faces the FDAE is viewpoint dependant, however (Hills et al., 2008, 2010a) argue that their results show something on the contrary. They found that 44% of the FDAE found by Benton et al. (2006) is actually viewpoint-invariant. In support of a viewpoint-invariant argument, Welling et al. (2009) found adapting to one view of an unfamiliar face with a raised mouth position caused a different view of the same face to be perceived as having a mouth looking lower than it was. After prolonged adaptation, the FDAE is partially viewpoint-independent in unfamiliar faces (Fang et al., 2007). In familiar faces, however, the FDAE is much more viewpoint-independent (Carbon et al., 2007).

Jiang et al. (2007) specifically tested the degree of familiarity that participants’ have with a face and the magnitude of the face identity aftereffect (FIAE, that Hills and Lewis, 2012 argue is an analog of the FDAE). Jiang et al. also tested aftereffects following within- and between-viewpoint adaptation. They trained 90 participants on a set of 16 faces to varying degrees of familiarity. Familiarity was manipulated by presenting the images a different number of times and in different viewpoints. Jiang et al. found that the magnitude of adaptation was greater for within-viewpoint adaptation. However, there was still significant adaptation for between-viewpoint adaptation. Moreover, the largest aftereffects were observed for the most familiar faces. Indeed, the difference between the FIAE to same- and different-viewpoint adaptation was smallest for the extremely familiar condition. Similarly, researchers have found that these aftereffects are very short-lived when testing unfamiliar faces, but can last over 24 h in familiar faces (Carbon and Leder, 2005, 2006). Differences in the transfer of aftereffects across viewpoints in familiar and unfamiliar faces have been attributed to differential representations (c.f., Megreya and Burton, 2006; Ryu and Chaudhuri, 2006): the representation of familiar faces is based on viewpoint-invariant coding (potentially three-dimensional), whereas the representation of unfamiliar face is based on pictorial coding (two-dimensional).

The aftereffects transfer across different images of one unfamiliar face to another (Webster and MacLin, 1999) and of one familiar face to another (Carbon et al., 2007). However, these authors have not assessed whether the aftereffects transfer from a familiar face to an unfamiliar face. If the aftereffect does transfer across faces of different levels of familiarity, then this would provide strong evidence for the norm-based coding theories (Leopold et al., 2001) of face memory. This would provide evidence for rapid updating of the face prototype (Carbon and Leder, 2005). However, familiar faces are seen to have a more robust representation and thus should be somewhat impervious to aftereffects caused by adaptation in unfamiliar faces.

### Personal familiarity

Herzmann et al. (2004) studied reaction time, priming, and skin conductance response when participants were presented with personally familiar faces compared to famous and unfamiliar faces. Reaction time responses were faster to personally familiar and famous faces than unfamiliar faces and the skin conductance response was greater for the familiar faces than unfamiliar faces. Additionally, personally familiar faces produced similar cognitive effects to famous faces. The similar results between personally familiar and famous faces could be due to how familiar the personally familiar faces were. The stimuli they used were university lecturers which could be argued do not represent the personally familiar category as well as perhaps parents or siblings would. It is unclear, whether personally familiar faces would produce different aftereffects to familiar faces.

There is some evidence from brain imaging to suggest that personally familiar faces are represented differently to other classes of familiar faces. Taylor et al. (2009) has found that the neurological response to personally familiar, famous, and unfamiliar faces is indeed different. The presentation of personally familiar faces activated more regions of the brain than unfamiliar faces. Presenting images of the participants’ parents caused a bilateralized activation of the cingulate gyrus, generally thought to be a multimodal processor (Turak et al., 2002). It is presumed to play a role in the integration of incoming sensory information perceived from the face (Devue and Brédart, 2007). While fewer brain regions were recruited for the processing of unfamiliar and famous faces than personally familiar faces, there were also some clear distinctions in the recruitment of the Fusiform Face Area (FFA; an area of the brain thought to be involved primarily with face perception). Taylor et al. found that personally familiar faces recruited the FFA bilaterally, whereas famous faces only activated the right-FFA. The processing of unfamiliar faces, on the other hand, appeared to recruit primarily the left hemisphere. Eger et al. (2005) have found similar results: greater response in the right-FFA when comparing famous faces with unfamiliar faces. In addition, famous faces cause greater adaptation in the FFA than unfamiliar faces. These results indicate that the FFA may be the locus for the FDAE and is likely to produce larger aftereffects for familiar faces than unfamiliar faces. In addition, these results suggest that there may be some differences in the transference of aftereffects from familiar to unfamiliar faces: specifically, since famous faces are predominantly processed in the right hemisphere and unfamiliar faces are predominantly processed in the left hemisphere, it should not be possible to cause adaptation that transfers across these types of faces. However, since personally familiar faces are processed bilaterally, aftereffects should transfer from these to both famous and unfamiliar faces and the converse should also be true.

### The present research

The present study aimed to determine whether there are differences in the FDAE for unfamiliar, famous, and personally familiar faces in terms of the magnitude of the aftereffects. Furthermore, we aim to assess whether the aftereffects can transfer across faces of differing levels of familiarity. Thus, participants were adapted to a distortion in either an unfamiliar, famous, or personally familiar face and the magnitude of the aftereffect was assessed in unfamiliar, famous, and personally familiar test images. All participants viewed the same test images which had all been distorted by moving the eyes either further or closer to the mouth. We assessed whether the more distorted faces appeared undistorted following the adaptation in a method similar to McKone et al. (2005). This technique allows us to see how adaptation affects participants’ subjective ratings of distortion and is analogous to participants perceiving a previously undistorted face as distorted following adaptation but allows for more trials.

## Method

### Participants

An opportunity sample of 48 (18 male) White British participants volunteered for this experiment as part of a course requirement. None of the participants knew each other (or each others’ parents). They had a mean age of 23.6 years (ranging from 18 to 33 years) and self-reported they had normal or corrected to normal vision. All participants were psychology undergraduates, studying at Anglia Ruskin University.

### Materials

One unfamiliar, one famous, and one personally familiar face (per participant) were used. These were matched for age, gender, image size, quality, and pose as best as possible. All poses were frontal and expressionless. Unfamiliar and personally familiar faces were provided by the participants who were instructed to obtain a picture of their parent which was expressionless, full frontal headshot, in front of a plain, light background, wearing a white shirt, using the best quality camera available. Each participant was tested on their own parent’s face and one of the other participants’ parent’s faces (thus, the stimuli were approximately matched across age) in addition to a famous face (this was also matched for approximate age). When submitting a photograph of a parent, participants were asked if they were familiar with a number of famous faces in order to ensure that an adequate level of superficial familiarity with the famous faces was maintained.

**Figure 1:**
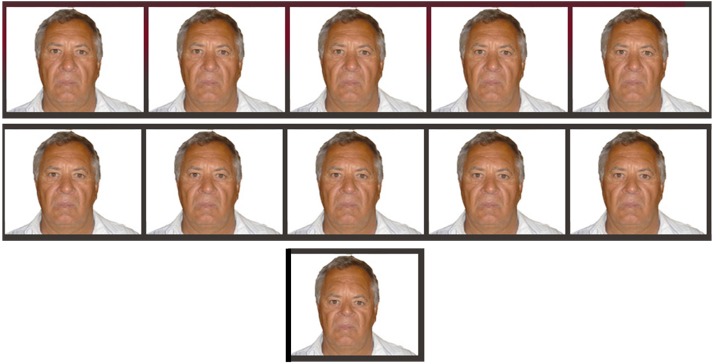
**Running from left to right, a complete set of images for a negatively distorted parent including test images ranging from −1 through to −10 as well as the adaptor image of −25**.

All pictures were then adjusted in Adobe Photoshop 7.0 so that the distance from the camera appeared the same. In addition, all backgrounds were masked out and matched. Each picture had a resolution of 96 dpi and the dimensions were constrained to 550 × 640 pixels (subtending visual angle 13.68° x 16.01°). Root mean contrast was kept constant across all stimuli by adjusting the brightness and contrast functions in Adobe Photoshop 7.0. They were then distorted by shifting the eyes up or down (see Hills et al., 2010b for a description of this procedure). Ten images shifted the eyes closer to the mouth by one pixel increments, producing images −1 through to −10, 10 images shifted the eyes further from the mouth, producing images +1 through to +10, and two extreme images were created (−25 and +25) in order to act as the adaptor stimuli. See Figure 1 for an example of the stimuli used in this experiment. This shifting technique has been used in a number of studies that also use a configural manipulation of the facial stimuli (McKone et al., 2005). All images could still be identified as belonging to the individual from which the distorted images were created. The images were displayed on a high resolution 17″ (1280 × 1024) LCD color monitor using MatLab in a quiet dimly lit research laboratory.

### Design

Using a 3 × 3 mixed design, the number of images perceived to be distorted was measured for three different test-image types (unfamiliar, famous, and personally familiar; within-subjects) for the different adaptors (unfamiliar, famous, and personally familiar; between-subjects). Even though participants were assigned to either a positive or negative adaptor-type this had no bearing on the results and so direction of the distortion is not considered a variable. Participants were randomly placed in groups, with the condition that there were an equal number of participants in each group. The order of image presentation was randomized.

### Procedure

The experiment was conducted in two stages: pre- and post-adaptation. Participants were assigned to view either positively or negatively distorted adaptors. If a negative adaptor had been assigned, all the stimuli throughout their entire experiment were negatively distorted. Similarly, if participants had been assigned to a positive condition all the stimuli were positively distorted.

#### Stage 1: Baseline

Participants were presented with the 10 distorted images from each of the types of faces (unfamiliar, famous, and personally familiar faces) that were distorted in the direction the participant had been assigned to. Each distorted face was shown 10 times each producing 300 trials. Participants were instructed to look at each image and judge whether it was “odd” or not (similar to McKone et al., 2005; Hills et al., 2010b). They were told some of the pictures were distorted and some were not. If they thought an image was normal they were asked to press the “M” key whereas if they thought the image was “odd-looking” they were asked to press the “Z” key. The face was on screen until participants responded. Preceding each face, a fixation cross appeared for 300 ms in the center of the screen.

#### Stage 2: Adaptation task

Participants were then told they would first see an adaptor face for 1 min. This appeared in the center of the screen. Following this, there was a repeat of the baseline phase, except that preceding each test face, the adaptor face was presented for 4 s but at twice the size and shifted 50 pixels into one of the four quadrants of the screen. This was done to control for lower-level visual based adaptation that is observed in the FDAE – data from Hills et al. (2010a) that indicate face aftereffects are approximately 50% low-level, image-based and 50% that is potentially higher-level (see, e.g., Rooney et al., 2012). The position of the adaptor face was randomized across trials.

## Results

**Figure 2:**
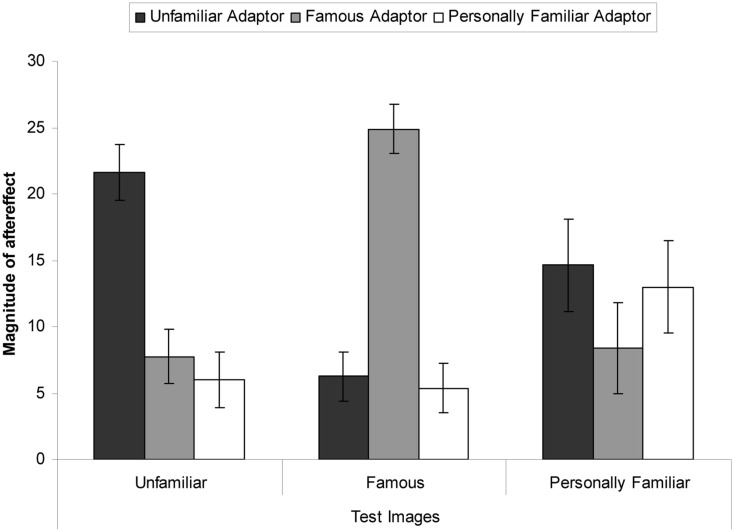
**Magnitude of aftereffect (mean number of test faces perceived to be distorted post-adaptation subtracted from the mean number of faces perceived to be distorted at baseline), for each image-type for every adaptor type**. Error bars show standard error.

The number of faces rated as distorted post-adaptation was subtracted from the pre-adaptation baseline test phase. Perceiving relatively less distortion during test means that the adaptor nullified the perceived distortion present in the test image, resulting in a greater aftereffect magnitude score. Perceiving relatively more distortion during test means that the adaptor did not affect the perceived distortion present in the test image as much, resulting in a smaller aftereffect magnitude score. The means are presented in Table 1 and Figure 2. These results indicate that aftereffects were observed in all conditions. However, when the test stimuli matched the adaptor the aftereffect was of a larger magnitude than when it was not. Similarly, the aftereffects were larger when the adaptor was unfamiliar than when the adaptor was personally familiar or famous.

**Table 1:** **Mean number of test faces perceived to be distorted post-adaptation subtracted from the mean number of faces perceived to be distorted at baseline, for each image-type for every adaptor type**.

		Test stimuli		
		Unfamiliar	Famous	Personally familiar
Adaptor type	Unfamiliar	21.63** (10.88)	6.25* (6.23)	14.63** (17.24)
	Famous	7.75* (6.44)	24.88** (10.97)	8.38* (4.87)
	Personally familiar	6.00* (6.61)	5.38* (2.42)	13.00** (16.00)

All aftereffects were significantly greater than zero, as revealed by nine Bonferroni corrected one-sample *t*-tests (*p < 0.05 and **p < 0.001). Standard deviation is presented in parentheses.

These data were subjected to a 3 × 3 mixed-subjects ANOVA. This revealed a significant interaction, *F*(4, 90) = 12.38, MSE = 117.90, *p* < 0.001, ${\text{η}}_{p}^{\text{2}}$ = 0.36. Bonferroni corrected simple effects showed that when the adaptor was unfamiliar, larger aftereffects were observed in the unfamiliar test images than the famous test images (mean difference = 15.38, *p* < 0.001) but not when the test images were personally familiar (mean difference = 7.00, *p* = 0.262). When the adaptor was famous, larger aftereffects were observed when the test images were famous than when they were unfamiliar (mean difference = 17.13, *p* < 0.001) or when they were personally familiar (mean difference = 16.50, *p* < 0.001). When the adaptor was personally familiar, marginally larger aftereffects were observed for personally familiar test images than famous test images (mean difference = 7.00, *p* = 0.046) and unfamiliar test images (mean difference = 7.63, *p* = 0.062).

There was also a significant main effect of adaptor type, *F*(2, 45) = 5.75, MSE = 31.28, *p* = 0.006, ${\text{η}}_{p}^{\text{2}}$ = 0.20. Dunnett *post hoc* tests were conducted, with the unfamiliar faces as the reference category. These revealed that when the adaptor was unfamiliar greater aftereffects were observed than when the adaptor was personally familiar (mean difference = 6.04, *p* = 0.007) but not when the adaptor was famous (mean difference = 0.50, *p* = 0.954). The main effect of test-image type was not significant, *F*(2, 90) = 0.02, MSE = 117.90, *p* = 0.985, ${\text{η}}_{p}^{\text{2}}$ = 0.01.

The mean number of faces perceived to be distorted at baseline ranged between 46.13 and 50.13 and there were no significant differences across any of the conditions (all *p*s > 0.80).

## Discussion

These results show an interesting and somewhat unexpected pattern of results. Firstly, the magnitude of the FDAE was typically greatest when the test images matched the adaptor type (except when the test images were personally familiar in which case there was no difference in the magnitude of adaptation for personally familiar and unfamiliar adaptors). Secondly, the aftereffect transferred from all adaptor types to all test stimuli. Thirdly, the aftereffect was weakest following adaptation to personally familiar faces, however this may be a result of there being lower matched-image adaptation than in the other conditions. Fourthly, the aftereffects in the personally familiar test images did not differ across adaptor type as much as the other conditions: the aftereffects in personally familiar faces was actually greater in the non-familiarity-match conditions than in the non-familiarity-match conditions for the other test faces. Finally, excluding the preceding effects, the aftereffects transferred least across unfamiliar adaptors to famous test images and famous adaptors to unfamiliar test images. We shall attempt to interpret each of these findings in turn.

The first two results summarized (FDAE greatest when adaptor and test images matched and that there was always some transference of the aftereffect) actually indicates some form of low-level aftereffect. Arguably, this aspect is likely to be based on similar mechanisms to shape aftereffects (Suzuki and Cavanagh, 1998) rather than any face norm-based coding. We make this supposition because all faces have a similar shape, and while they were positioned in different areas of the screen (thus the aftereffects are non-retinotopic), they were of a similar magnitude. Similarly, the fact that the transfer of aftereffect was typically lower when the image changed than when it was the same suggests some low-level image-based adaptation. In addition, it seems likely that there would be no reason to engage higher-level cognitive processing when lower-level more general processes would suffice. We would expect to see the neural locus of these aftereffects to be somewhere in the occipital lobe, before the FFA (see below for further elucidation of this point).

The third finding, that the aftereffects following adaptation to personally familiar faces was smaller than following adaptation to other types of faces and that they were surprisingly small when the test images were personally familiar face suggests that the representations of personally familiar faces is more robust and stable than those of unfamiliar and famous faces. This could come as a surprise, since we have already stated that humans are experts at recognizing personally faces that they have not seen in some time and those faces are likely to change in that time. Indeed, every morning, your partner will look slightly different to the night before. This could lead us to hypothesize that the representation of personally familiar faces should update more easily than unfamiliar people and thus be more adaptable. However, perhaps the robustness of the representation means that personally familiar faces are less adaptable because we know that we see them in many different conditions and thus consider any variant of the face acceptable to the identity. Alternatively, extreme familiarity may cause participants to know that those faces can never be distorted in that way. In other words, because we have so much experience of a personally familiar face, we know the entire variability of their face therefore adaptation cannot cause us to see a distortion in the personally familiar face, that is not physically possible, because of the restrictions placed on the representation of that face.

The preceding argument is similar to one made by Hills et al. (2010b) in terms of how face-space might develop. They found that children could be adapted to facial distortions that adults could not be: specifically, if each eye was shifted in different directions adults did not show aftereffects whereas children did. Both adults and children showed similar aftereffects to possible distortions (both eyes shifted together). Hills et al. theorized that as children become more familiar with faces, the neural responses to facial distortions becomes restricted such that only possible distortions can be processed as a face. Thus, the neurons that processed an impossible configuration are pruned since they are no longer useful (e.g., O’Leary and Koester, 1993). This is only conjecture, but fits the pattern of data here to: as personally familiar faces are encountered so frequently, we know the entire range with which they can be distorted, and anything beyond that is not coded as a face. Therefore, it is harder to be adapted to distortions in personally familiar faces.

We also found that aftereffects in personally familiar test faces were of similar magnitude whether the adaptor was unfamiliar or personally familiar. This effect is highly interesting for it suggests that there is significant correlation in the representation of personally familiar and unfamiliar faces and this is somewhat greater than the correlation in the representation of personally familiar and famous faces. This could be related to the fact that aftereffects seem easier to produce in personally familiar test faces than unfamiliar faces and famous faces overall (if you exclude the familiarity-matched conditions). Thus, something about the representation of personally familiar faces is linked to both unfamiliar and famous faces.

The final two findings are related to the possible neural architecture of these aftereffects. If the main locus of the FDAE is the FFA, and we accept that familiarity affects the hemisphere of processing (Taylor et al., 2009), then these results seem quite clear. Given that famous faces are primarily processed in the right-FFA and unfamiliar faces are primarily processed in the left-FFA (Taylor et al., 2009), it would be difficult for the aftereffects to transfer across these types of faces. In other words, we would expect that the viewpoint-dependent aftereffects to be located in the left-FFA, but viewpoint-independent aftereffects to be located in the right-FFA. There would be little communication between the left- and right-FFAs during the processing of faces, so the aftereffects are unlikely to transfer across.

This then links on to the idea that personally familiar faces are represented bilaterally (Taylor et al., 2009). If this is the case, then the FDAE should transfer from unfamiliar and famous faces to personally familiar faces and vice versa. However, this transfer should be of a smaller magnitude than within hemisphere (within class of face) transference, because only part of the processing has been adapted. If an aftereffect is caused by adaptation in an unfamiliar face, then neurons in the left-FFA will become adapted, thus responses to a familiar face will be smaller than without this adaptation. However, because there has not been any adaptation to the right-FFA, this aftereffect will be smaller than if the representation had been bilateral. To explain some transference of the aftereffect in all conditions, we would suggest that there is some low-level adaptation occurring prior to the FFA. This low-level aftereffect is unlikely to be lateralized and is likely to occur in early visual processing areas.

The previous explanation seems to fit with all the data except the fact that aftereffects were greater in personally familiar faces following adaptation to distortions in unfamiliar faces. This may be linked to our theorizing for the third finding. If adaptation is harder to produce in personally familiar faces but aftereffects can transfer to personally familiar from both unfamiliar and familiar faces because of shared neural architecture then we have at least a partial explanation.

These results also inform us how the representation of familiar and unfamiliar faces may differ. Given the face-space (Valentine, 1991) model for face memory, it is assumed that all faces are stored within this multidimensional space. Evidence for norm-based coding comes from aftereffects changing the locus of the prototype (c.f., Leopold et al., 2001). We have suggested that it is, firstly, harder to change the prototype when adapting to a personally familiar face and, secondly, changing the prototype by adapting to a famous face does not affect the prototype for unfamiliar faces. To interpret these results within face-space, we outline four possibilities that could be suggested are cause for the above results.

(1) It may be that personally familiar faces are not stored in the face-space. They are so familiar that they are stored as a unique entity. (2) There may also be a face-space that is used for the coding of familiar faces (located in the right hemisphere) and one used for the coding of unfamiliar faces (located in the left hemisphere). These would be based on different prototypes. This would suggest that adapting to a particular personally familiar face would not cause any discernable change in the perception of an unfamiliar face in a typical face identity aftereffect paradigm. This hypothesis seems unlikely, given that the explanation for the face identity aftereffect is a simple shift in the perceptual space (c.f., Hulbert, 2001). (3) A more plausible explanation is that different dimensions of the face-space are represented in different hemispheres. The dimensions that are used to recognize famous faces are located in the right hemisphere (those representing internal features, Ellis et al., 1979) and the dimensions that are used to recognize unfamiliar faces are located in the left hemisphere (those representing external features). There may be direct communication between all the dimensions of the face-space, but when presented with a face only those dimensions that are relevant are actually used. (4) A final explanation is that unfamiliar faces are not actually faces at all (Megreya and Burton, 2006). Unfamiliar faces may actually be represented as objects and all aftereffects observed in unfamiliar faces are the result of shape aftereffects and have nothing to do with face-specific mechanisms.

It is clearly necessary that further research be conducted in order to answer the question of which of these models explains the present data (no face-space for personally familiar faces; two separate face-spaces; different dimensions for familiar and unfamiliar faces; or unfamiliar faces are not faces). It may be possible, for example, to create one type of aftereffect (say expansion) in famous faces and another type of aftereffect (say compression) in unfamiliar faces. If this result were possible, then it would indicate that there were two separate face-spaces for familiar and unfamiliar faces (c.f., Rhodes et al., 2004). To assess whether unfamiliar faces are not really processed as faces, it may be possible to explore aftereffects transferring from shapes and objects to faces (c.f., studies by Fang and He, 2005, on viewpoint aftereffects showing that this does not occur). If unfamiliar faces are not faces, then this transference should occur, but it should not occur for famous faces.

In conclusion, this study has provided further evidence for the distinct representations of unfamiliar, famous, and personally familiar faces. We have presented evidence for some image-based aftereffects and also some aftereffects that suggest different neural coding for unfamiliar, famous, and personally familiar faces. We have interpreted these findings within a neural architecture suggesting that these aftereffects are hemisphere specific.

## Conflict of Interest Statement

The authors declare that the research was conducted in the absence of any commercial or financial relationships that could be construed as a potential conflict of interest.
